# What the papers say

**DOI:** 10.1093/jhps/hnab053

**Published:** 2021-08-08

**Authors:** Ali Bajwa

**Affiliations:** The Villar Bajwa Practice, Princess Grace Hospital, London W1G6PU, UK

The *Journal of Hip Preservation Surgery* (*JHPS*) is not the only place where work in the field of hip preservation can be published. Although our aim is to offer the best of the best, we are continually fascinated by work that finds its way into journals other than our own. There is much to learn from it, and so *JHPS* has selected six recent and topical subjects for those who seek a summary of what is taking place in our ever-fascinating world of hip preservation. What you see here are the mildly edited abstracts of the original articles, to give them what *JHPS* hopes is a more readable feel. If you are pushed for time, what follows should take you no more than 10 min to read. So here goes …

## ANTEROINFERIOR HIP INSTABILITY IN FLEXION DURING DYNAMIC ARTHROSCOPIC EXAMINATION IS ASSOCIATED WITH ABNORMAL ANTERIOR ACETABULAR HORN

The authors from Baylor University, TX [1] state that the stabilization of the femoral head is provided by the distal acetabulum when the hip is in a flexed position. However, the osseous parameters for the diagnosis of hip instability in flexion are not defined.

They have attempted to determine whether the osseous parameters of the distal acetabulum are different in hips demonstrating anteroinferior subluxation in flexion under dynamic arthroscopic examination, compared with individuals without hip symptoms. Their hypothesis was that the morphometric parameters of the anterior acetabular horn are distinct in hips with anteroinferior instability, compared with asymptomatic hips.

In this case-control study, a total of 30 hips with anteroinferior instability in flexion under dynamic arthroscopic examination were identified. A control group of 60 hips (30 patients), matched by age and sex, was formed from individuals who had undergone pelvis magnetic resonance imaging (MRI) for non-orthopaedic reasons. Unstable and control hips were compared according to the following parameters, assessed on axial MRI scans of the pelvis: anterior sector angle (ASA), anterior horn angle (AHA), posterior sector angle (PSA), posterior horn angle (PHA), acetabular version, lateral centre-edge (CE) angle, acetabular inclination (Tönnis angle) and femoral head diameter.

The authors found that the coverage of the femoral head by the anterior acetabular horn was decreased in unstable hips, compared with the control group (mean ASA, 54.8° versus 61°, respectively). Unstable hips also had a steeper anterior acetabular horn, with an increased mean AHA compared with controls (52.5° versus 46.8°, respectively). An ASA <58° had a sensitivity of 0.8, a specificity of 0.68, a negative predictive value of 0.87 and a positive predictive value of 0.56 for anteroinferior hip instability. An AHA >50° had a sensitivity of 0.77, a specificity of 0.72, a negative predictive value of 0.86 and a positive predictive value of 0.57 for anteroinferior hip instability. There was no statistically significant difference in the mean PSA, PHA, acetabular version, lateral CE angle, acetabular inclination or femoral head diameter between unstable hips and controls.

Therefore, they concluded that the abnormal morphology of the anterior acetabular horn is associated with anteroinferior instability in hip flexion. The ASA and AHA can aid in the diagnosis of hip instability.

## SUBSPINE HIP IMPINGEMENT: CLINICAL AND RADIOGRAPHIC RESULTS OF ITS ARTHROSCOPIC TREATMENT

In this study, Roos *et al.* [2], from Brazil, evaluated the clinical and radiographic results, as well as complications related to patients undergoing arthroscopic treatment of subspine hip impingement. They retrospectively evaluated 25 patients (28 hips) who underwent arthroscopic treatment of subspine impingement between January 2012 and June 2018. The mean follow-up was 29.5 months, and the patients were evaluated clinically by using the Harris hip score modified by Byrd (MHHS), the non-arthritic hip score (NAHS), and in terms of internal rotation and hip flexion. In addition, they analysed the imaging examinations for the CE acetabular angle, the Alpha angle, the presence of posterior wall sign, the degree of arthritis, the presence of heterotopic hip ossification and the Hetsroni classification for subspine impingement.

They reported a mean postoperative significant increase of 26.9 points for the MHHS, 25.4 for the NAHS, 10.5° in internal rotation and 7.9° for hip flexion. As for the radiographic evaluation, the authors found an average reduction of 3.3° in the CE angle and of 31.6° for the Alpha angle. Eighteen cases (64.3%) were classified as Grade 0 osteoarthritis of Tönnis, and 10 (35.7%) were classified as Tönnis Grade 1. Two cases (7.1%) presented Grade 1 ossification of Brooker. Most hips (*n *=* *15, 53.6%) were classified as Type-II subspine impingement of Hetsroni *et al.*

The authors thus concluded in this study that patients undergoing arthroscopic treatment with subspine impingement show improvement in clinical aspects and radiographic patterns measured postoperatively, at a mean follow-up of 29.5 months.

## MACHINE LEARNING ALGORITHMS PREDICT CLINICALLY SIGNIFICANT IMPROVEMENTS IN SATISFACTION AFTER HIP ARTHROSCOPY

The authors [3] from IL, United States, aim to develop machine learning algorithms to predict failure to achieve clinically significant satisfaction after hip arthroscopy.

They queried a clinical repository for consecutive primary hip arthroscopy patients treated between January 2012 and January 2017. Five supervised machine learning algorithms were developed in a training set of patients and internally validated in an independent testing set of patients by discrimination, Brier score, calibration and decision-curve analysis. The minimal clinically important difference (MCID) for the visual analogue scale (VAS) score for satisfaction was derived by an anchor-based method and used as the primary outcome.

The authors included a total of 935 patients, of whom 148 (15.8%) did not achieve the MCID for the VAS satisfaction score at a minimum of 2 years postoperatively. The best-performing algorithm was the neural network model (C statistic, 0.94; calibration intercept, −0.43; calibration slope, 0.94; and Brier score, 0.050). The five most important features to predict failure to achieve the MCID for the VAS satisfaction score were history of anxiety or depression, lateral CE angle, preoperative symptom duration exceeding 2 years, presence of 1 or more drug allergies and Workers’ Compensation.

In conclusion, the authors report that supervised machine learning algorithms conferred excellent discrimination and performance for predicting clinically significant satisfaction after hip arthroscopy, although this analysis was performed in a single population of patients. They felt that external validation was required to confirm the performance of these algorithms.

## PREVALENCE OF FEMOROACETABULAR IMPINGEMENT IN NON-ARTHRITIC PATIENTS WITH HIP PAIN: A META-ANALYSIS

In this meta-analysis, Jauregui *et al.* [4] explore the prevalence of femoroacetabular impingement syndrome (FAIS) in symptomatic patients who lack evidence of hip osteoarthritis (OA). The purpose of this study was to calculate the prevalence of FAIS in this patient population.

They reviewed the libraries of PubMed, Embase and Ovid systematically for all studies between 2009 and 2019, investigating femoroacetabular impingement (FAI) and hip pain. Level I–IV studies delineating patients with hip pain, who do not have OA (Tönnis or Outerbridge grades <3) were included. Demographics, outcomes, radiographic parameters and criteria were entered into a meta-analysis to calculate the incidence of FAIS in non-arthritic symptomatic hips.

In total, 2264 patients (2758 hips) were included in the pooled analysis. The weighted mean age was 31 years. They found that the incidence of FAIS in patients with no evidence of osteoarthritis but who complain of hip pain was 61% (47.3–74.4%). In total, 1483 hips were diagnosed with FAIS. Of the studies that described, the rates of all three of the various subtypes of FAIS in their reports, 37% had a combined type, 38% had a cam-type and 25% had a pincer-type FAIS.

The authors concluded that the FAI should be suspected in 47–74% of patients with hip pain and without arthritis. Physicians must maintain a high index of suspicion for FAIS in young patients presenting with hip pain, as FAIS is a common and treatable condition that, if left alone, may lead to hip degeneration.

## DIFFERENCES IN CLINICAL PRESENTATIONS AND SURGICAL OUTCOMES OF GLUTEUS MEDIUS TEARS BETWEEN MEN AND WOMEN

The authors [5] from the United States, in this cohort study, report that gluteus medius (GM) tears often occur in women aged >50 years. There is a paucity of literature comparing sex-based differences in those undergoing GM repair. Their aim was to explore differences between women and men in clinical presentations and patient-reported outcome (PRO) scores at a minimum 2-year follow-up after undergoing GM repair.

Data were prospectively collected and retrospectively reviewed. All included patients had postoperative scores for the following PROs: modified Harris Hip Score (mHHS), NAHS, Hip Outcome Score-Sports Specific Subscale (HOS-SSS) and International Hip Outcome Tool-12. Men were propensity score-matched 1:3 to women according to concomitant arthroscopic procedures and follow-up time. Clinical effectiveness was determined through a uniquely calculated MCID, for the mHHS and NAHS specific to this study population.

The authors reported that 13 men were successfully propensity score-matched to 39 women. Women and men were a mean of 55.87 and 62.38 years old, respectively. Men were at a significantly increased risk for associated lumbar pathology as compared with women (relative risk, 3.32). Women showed significant improvement from preoperative to minimum 2-year follow-up for the mean mHHS (59.32–83.81), NAHS (56.23–83.78), HOS-SSS (33.35–67.88) and VAS (5.48–1.93). Similarly, men showed significant improvement for the mean mHHS (63.50–84.77), NAHS (61.52–84.42), HOS-SSS (33.97–63.62) and VAS (4.93–1.86). The MCIDs for the mHHS and NAHS were calculated to be 7.89 and 7.24, respectively. Of the women, 28 (72%) and 34 (87%) met the MCID for the mHHS and NAHS. Eleven (85%) men met the MCID for the mHHS and NAHS.

In their conclusion, the authors remarked that women and men can both benefit after GM repair. Men were older and had an increased risk for associated lumbar pathology than women at the time of surgery. Men and women both experienced significant improvements in PROs and compared favourably in terms of clinical effectiveness at a minimum 2-year follow-up.

## TIME TAKEN TO RESUME DRIVING FOLLOWING HIP ARTHROSCOPY

The authors [6] from South Korea aimed to answer the importance of return-to-driving after hip arthroscopy, which is a common concern among patients undergoing the procedure. Their study specifically assessed whether the patients who had undergone right hip arthroscopy presented with poorer driving performance than the patients with normal hips and analysed the time required to regain preoperative driving performance.

They included 47 patients, who had undergone right hip arthroscopy and consented to their test protocol in the study. Using an immersive driving simulator, the patients were tested for their brake reaction time (BRT), total brake time (TBT) and brake pedal depression (BPD) preoperatively and postoperatively. The first postoperative assessments were conducted when the patients could comfortably sit on the driving seat, and the follow-up assessments were conducted for six consecutive weeks at weekly intervals. The patients were divided into the following two groups based on the type of surgery that they underwent: the FAI surgery group and the simple hip arthroscopy (SA) group. Twenty healthy volunteers underwent driving assessments thrice, at weekly intervals, and constituted the control group. The braking parameters were compared between preoperative and postoperative measurements and among the FAI surgery, SA and control groups.

The preoperative braking parameters of the patients who underwent arthroscopy did not differ significantly from those of the controls (*P* = 0.373, 0.763 and 0.447 for the BRT, TBT and BPD, respectively). All braking parameters returned to normal in 2 weeks in the FAI surgery group and in 1 week in the SA group.

The authors thus concluded that the driving performance of patients who underwent right hip arthroscopy is comparable to that of individuals with normal hips and that the braking parameters may normalize to the preoperative state at 1 week after SA and 2 weeks after FAI surgery.

## CONFLICT OF INTEREST STATEMENT

None declared.
